# Mapping evidence on youth experiences and practices on partner notification for sexually transmitted infections in sub-Saharan Africa: scoping review

**DOI:** 10.3389/frph.2026.1729464

**Published:** 2026-04-20

**Authors:** Makananelo Kenoakae Pule, Raikane James Seretlo, Oluwafemi Omoniyi Oguntibeju, Thabo Ishmael Lejone, Mathildah Mpata Mokgatle

**Affiliations:** 1 Department of Public Health, Sefako Makgatho Health Sciences University, Pretoria, South Africa; 2 Department of Biomedical Sciences, Faculty of Health and Wellness Sciences, Cape Peninsula University of Technology, Bellville, South Africa; 3 Department of Clinical Research, Division of Clinical Epidemiology, University Hospital Basel, Basel, Switzerland

**Keywords:** partner notification, sexually transmitted infections, sub-Saharan Africa, symptoms of diseases, youth

## Abstract

**Introduction:**

Partner notification (PN) involves informing current and past sexual partners of their potential exposure to sexually transmitted infections (STIs) and encouraging them to seek testing and treatment. Effective PN is critical for STI control, yet little is known about how youth in sub-Saharan Africa experience and practice PN. This paper reports on a mapping of evidence on youth-related PN experiences, practices, barriers, and facilitators in sub-Saharan Africa.

**Methods:**

A scoping review was conducted using the Arksey and O'Malley framework, complemented by the Joanna Briggs Institute methodology for scoping reviews. Evidence published between January 2016 and January 2026 was searched on PubMed, SCOPUS, Medline, CINHAL and OVID. The database search took place in February 2026. The terms used for review included (“youth” OR “adolescent” OR “young people” AND “partner notification” OR “contact tracing” AND “sexually transmitted infection” OR “STI” AND “sub Saharan africa” OR “sub-Saharan countries”). Screening of titles and abstracts, and data extraction were conducted independently; discrepancies were resolved by the third reviewer.

**Results:**

A total of 117 articles were identified with 59 duplicates records removed. Eighty-two records titles and abstracts were screened, and 69 articles were excluded. Ten studies proceeded to full-text screening, of which three of them met eligibility criteria for inclusion and were retained for analysis. The three articles were from Southern Africa and were youth focused, covering the ages 15–24 years. Two of the studies were conducted in Zimbabwe, followed by South Africa (*n* = 1). The study designs included cohort study (*n* = 1), mixed method (*n* = 1) and qualitative intervention design (*n* = 1). The barriers to PN were identified as centering around factors relating to health, infection, and social dynamics.

**Conclusion:**

Partner notification remains a critical strategy for STI control. However, the review reflected that PN remains difficult among youth. Youth's partner notification is affected by patient related factors, sociocultural factors and health system factors.

**Scoping Review Registration:**

https://doi.org/10.17605/OSF.IO/8972W.

## 1 Introduction

The World Health Organization (WHO) identifies human immunodeficiency virus (HIV), viral hepatitis and sexually transmitted infections (STIs) are major epidemics that prevent progress toward global health targets (1). Globally, an estimated 374 million new STI cases occur annually (2). Sub-Saharan Africa continues to face a significant burden of STIs, accounting for an estimated 93 million new cases each year (2–4). Sexually transmitted infections prevalence remains high among populations such as adolescent girls and young women, and incarcerated people (3). In developing countries, STI management relies primarily on syndromic approaches, whereby treatment is based on symptoms presented at the health facility (2, 3). This syndromic management approach often results in delayed detection of infections, continued transmission between sexual partners, missed opportunities for prevention, and the potential for clinicians to over or undertreat symptomatic patients (5–7).

A comprehensive package of sexually transmitted infection prevention services must include health education, screening for STIs by health care workers, providing PN services, and managing sexually transmitted infections (1). Sexually transmitted infection services must be offered to clients as part of range of options based on their needs and preferences within a comprehensive package of voluntary STI testing, prevention and care (8). Partner notification is the process of informing sexual partners of potential risk of exposure to STIs (8). When implemented effectively, PN can reduce reinfection, disrupt STI transmission, and support the early detection of STIs amongst partners (5, 9). However, its effectiveness may be limited by fear, violence, blame, health system factors, stigma and health system related factors like trained health care workers (HCW) and reliable diagnostic methods (10, 11). A study conducted in Zimbabwe examining youth experiences of PN following the STI diagnosis found that although 41.2% (745/1807) received PN slips, only 5.7% of their partners subsequently sought treatment services (12). Other studies conducted in sub Saharan Africa have shown that type of partner, relationship duration, the notification preference, communication about STI among partners, contact information and discussion about STI exposure among partners play a role on effective implementation of PN (10, 11). While PN has been studied among pregnant women, general population, key populations (11, 13, 14), there is limited research focusing exclusively on experiences and practices of youth in sub-Saharan Africa in this regard. Young people face unique challenges and specific vulnerabilities regarding contracting STIs (15). Multiple factors contribute to risky behavior by youth, such as early sexual debut, the age of sexual partners, multiple partners, casual partners, inconsistent use of condoms and perceptions of low risk to be infected by STIs or HIV (15–17). Additionally, barriers that youth face are amplified by their limited access to health facilities that could provide youth-responsive sexual and reproductive services (5).

Partner notification remains a critical strategy in STI control because it facilitates early diagnosis among sexual partners and promotes behavior modification for both clients and their sexual partners (10). Furthermore, PN contributes to reducing the spread of STIs in communities (10, 16). However, youth face challenges in notifying partners, and they cite various reasons (10, 12). Existing literature highlights the need to examine whether PN is a suitable approach for youth; interventions that balance risks and protect youth during engagement of care must be examined (10, 12).

Several PN approaches exist, including delayed provider referral, enhanced patient referral, expedited partner referral, patient referral, provider assisted referral, provider patient referral and social network approaches (8, 18, 19). In delayed provider referral, where the patient diagnosed or with possible STI gets into contract with the HCW to disclose the STI diagnoses or exposure to their sexual partner within a specified period. If the partner does not disclose at agreed timeframe, HCW communicate with the partner directly and offers STI testing (5, 8). With enhanced patient referral, the trained HCW uses multiple support tools to support the patient disclose to their partners. Tools maybe a written information, leaflets, card, digital tool, and collection of STI self-test kit (8, 15, 20) The use of digital platforms in PN is utilized during provider-initiated method; whether sexual partners accept the communication is ambiguous (12). Another is expedited partner therapy (EPT) enables patients to deliver the medications directly to their partners without the need for facility visits (18). In provider referral, HCW directly notify to partners of their exposure STI exposure (11), provider patient referral involves HCWs accompanying or offering support during disclosure (8). The social network approach is whereby trained HCW request the STI diagnosed patient with high ongoing risk to contract STI to invite their sexual and or individuals in their social network to seek STI testing services (8).

The study among youth in South Africa comparing university students and out of school youth found that 67% of university students preferred short message service (SMS) notification compared with 42% of out of school youth. Partner notification slips were preferred by fewer young people overall (15). Literature identifies no single approach that is suitable for all populations; services may need to be customized for different segments of populations (21). In a study conducted among youth in Zimbabwe to understand their experiences of PN, PN slip uptake was 41.2% (12). Although in Africa, the most common PN approaches are patient initiated, and provider initiated or assisted methods (10–12, 15, 21). There is limited evidence published amongst youth aged 15–24 years on their experiences and practices on PN for sexually transmitted infections. To inform the development of youth-friendly PN strategies for the control of curable sexually transmitted infections in Maseru, Lesotho. We mapped evidence on youth-related PN experiences, practices, barriers, and facilitators in sub-Saharan Africa.

## 2 Scoping review objectives

The objectives of the scoping review were as follows:

- To identify studies that explored youth experiences of PN to control curable STIs in sub-Saharan Africa;
- To examine the practices of youth regarding PN to control curable STIs in sub-Saharan Africa;
- To examine reactions and dynamics related to disclosing STI infections to sexual partners in sub-Saharan Africa;
- To explore barriers and facilitators for effective PN by youth; and
- To expose knowledge gaps that should be taken into consideration in developing tailored partner notification strategies for youth in sub-Saharan Africa.

## 3 Scoping review questions

The scoping review was set up to answer the following research questions:

- What are the experiences of youth regarding PN services to control curable STIs in sub-Saharan Africa?
- What are the practices of youth regarding PN services to control curable STIs in sub-Saharan Africa?
- What are the reactions and dynamics related to disclosing STI infections to sexual partners in sub-Saharan Africa?
- What are the barriers to and facilitators of effective PN of exposure to STIs by youth in sub-Saharan Africa?
- What are the knowledge gaps on PN strategies for youth in sub-Saharan Africa?

## 4 Rationale of the review

Globally, STIs remain a public health challenge. In addition to biomedical interventions, PN is a critical approach to preventing STIs (1).

### 4.1 What is already known?

Partner notification is a way to reach sexual partners at risk of acquiring STIs and preventing STI transmission; however, among youth there are challenges that minimize the benefits of partner identification (29).

### 4.2 What evidence does the study contribute?

The review documents a substantial evidence gap on PN experiences and practices targeting youth aged 15–24 years. The youth aged 15–24 years, though less researched group, have unique needs, vulnerabilities and dynamics surrounding partnerships that require tailored services.

## 5 Methodology

### 5.1 Design

The scoping review is the first phase of a study that obtained ethical clearance and approval from Sefako Makgatho Health Sciences University Ethics Committee (SMUREC/H/32/2025:PG) and Lesotho Ethics Committee (ID177-2025).

The scoping review protocol was registered on Open Science Framework under trial registration number https://doi.org/10.17605/OSF.IO/8972W.

**Figure 1:**
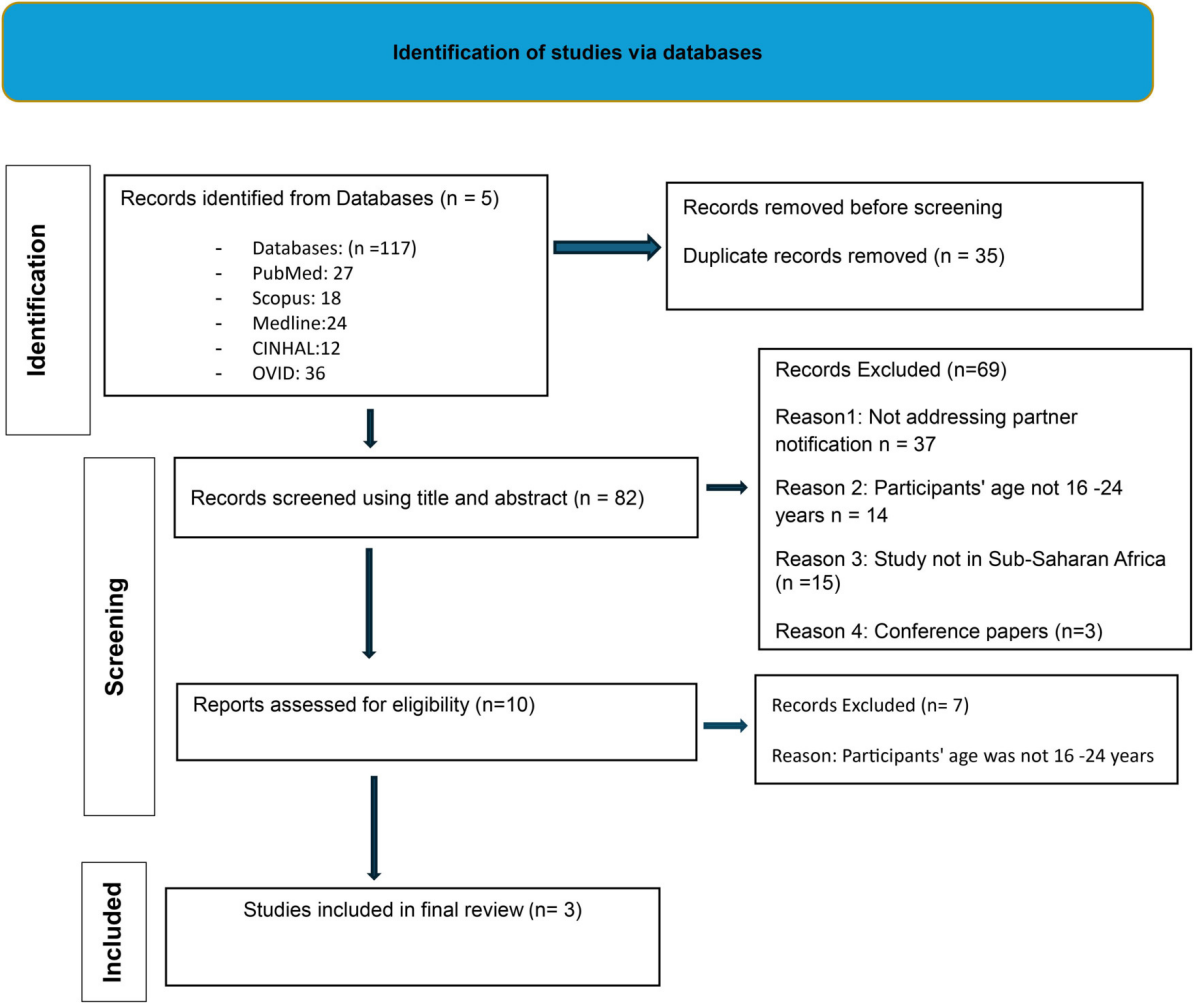
Scoping Review: Youth Experiences and Practices on Partner Notification for Sexually Transmitted Infections in sub-Saharan Africa.

While the scoping review was registered to cover curable STIs and the region of Africa, review of the context and the scope evolved to cover STIs in sub-Saharan Africa. We conducted the scoping review by following the Joanna Briggs Institute's methodology for scoping reviews. The review is reported in accordance with Preferred Reporting Items for Systematic Review and Meta-Analysis extension for Scoping Reviews (PRISMA-ScR) guidelines (15–17) (Supplementary Appendices A, B—Figure 1 show the process followed in the scoping review).

### 5.2 Search strategy

We performed an electronic database search on Pubmed, Scopus, Medline, CINAHAL, and OVID from February 2026. The terms used included “youth” OR “adolescent” OR “young people” AND “partner notification” OR “contact tracing” OR “partner referral” AND “sub Saharan africa” OR “subsaharan countries”. An experienced librarian supported the scoping review by piloting the search strategy before the full search of five databases was conducted. The search approach balanced comprehensiveness with precision by including and exploring the general index term as sexually transmitted infection. Detailed search terms are listed (Supplementary Appendix C). The searched studies were uploaded on Rayyan software for screening (22).

#### 5.2.1 Eligibility

##### 5.2.1.1 Inclusion criteria

The topic of the studies included in the scoping review was STIs. Peer-reviewed articles published from January 2016 to January 2026 and focusing on sub-Saharan Africa were included in the review. Studies that focused on youth aged 15–24 years were included in the review.

##### 5.2.1.2 Exclusion criteria

Studies that had been conducted outside sub-Saharan Africa, which did not report on curable STI partner notification, those with participants age less than 15 years and greater than 24 years and manuscripts based on conference were excluded. Additionally, studies published before 2016 were not included in the review.

### 5.3 Selection process

Using Rayyan software, three independent reviewers (MKP, RS and TL) screened articles at the title, abstract and full text levels (22). The reviewers met for discussion to resolve disagreements on the review. The key characteristics and key themes of selected articles that met inclusion criteria were charted in Microsoft Word (Supplementary Appendix D). The data extraction tool was used to summarize the study characteristics, study design, outcomes, and key findings.

## 6 Results

### 6.1 Search results

In total, 117 articles were identified from the five databases and were uploaded in Rayyan software. A total of 59 duplicates were detected and resolved. Then, 82 articles were independently reviewed by three reviewers at title and abstract levels. After reading articles and the titles, 69 articles were excluded because: (1) articles did not cover partner notification services, (2) study participants’ age was not 15–24 years, (3) articles were covering countries outside the sub-Saharan region, and lastly, they were conference paper. Ten studies proceeded to full-text screening, of which three of them met eligibility criteria for inclusion and were retained for analysis. The seven studies which were excluded from full text review was because the study's participants age was not 15–24 years of age.

### 6.2 Overview of articles included

A total of three articles published between January 2016 and January 2026 were included in the scoping review (Table 1). Two thirds of studies (*n* = 2) were conducted in Zimbabwe (12, 23). Another study had been conducted in South Africa (5). Studies reviewed involved cohort, qualitative study and mixed methods studies (5, 12, 23).

**Table 1:** Reviewed articles (*n* = 3) published 2016–2026.

Country	Studies	Population/Sample size	Study Design	Outcome details, recommendations for future research
Zimbabwe	Lariat et al. (12)	1,807	Mixed method	There is a need to interrogate whether PN is suitable for youth
		Young people aged 16–24 years		
	Mackworth-Young et al. (23)	15	Qualitative, intervention	The youth friendly services facilitate uptake of STIs services however research is required on socially safe partner notification strategies.
		Young people aged 16–24		
South Africa	Chitneni et al. (5)	216	Prospective cohort	PN by youth is challenging and novel strategies are required to overcome barriers
		Youth aged 16–24 years		

For this review, youth was defined as any individual aged from 15 to 24 years, regardless of gender (24). The studies included in the review focused on youth studies (5, 12, 23). Within the reviewed studies, sample sizes ranged from 15 participants in smaller qualitative, cohort and mixed method study to thousands of index clients and partners (5, 12, 23). The studies included in the review reflected diverse approaches to PN in sub-Saharan Africa. Studies reported on patient-initiated notification where they used referral slips or were encouraged to verbally inform their partners about their STI diagnosis (5, 12, 23). The studies included in the review measured different outcomes pertaining to PN. Outcomes can be categorized into experiences and practices of youth on PN, reactions and dynamics that affect PN, barriers to PN.

### 6.3 Experiences and practices of partner notification

Partner notification experiences varied widely shaped by relationship dynamics, age and context (5, 12). The study conducted amongst youth in South Africa reported that 74% of participants notified their partners about STI diagnosis. Common reasons for not notifying partners were embarrassment, fear of judgement by partner, possible dissolution of the relationship and participants not being aware that they had STI requiring partner's notification (5). In the study conducted in Zimbabwe, youth expressed fears of reputational harm, gossip and social judgement once information about their STI is revealed. This fear was coupled with a feeling of being unprepared for PN conversations and desired stronger counselling for participants to navigate partner's reaction and their safety (12).

The PN experiences on casual and steady relationship differed Participants who were in a steady relationship was likely to result in successful PN, whereas a casual, concurrent partnership or short-term partners did not have high disclosure rates. In casual relationship, STI diagnosed patients felt notifying partner was unnecessary, no commitment to notify and no emotional obligation (23).

Across the youth studies, PN was reported to be challenging, the motivation to notify partners was heightened by their experiences with health providers. Some of facilitators for notifying partners were: protecting health of a partner or preventing reinfection and ensuring that partner received STI treatment (12, 23).

In reviewed studies, common practices for PN was: patient referral through utilization of the referral slip or through verbal disclosure. A youth study conducted in Zimbabwe reported that the youth felt ill equipped when confronted with notifying their partners and the PN was framed as a moral obligation (12). Another study in Zimbabwe reported that practice of notifying partners by youth was influenced by the support that youth received from the health care workers (23).

### 6.4 Reactions and barriers to partner notification

The partner anticipated or actual reactions was deeply intertwined with barriers to notify partners about STI diagnosis. Frequently reported barriers were: fear of negative partner reactions, such as blame, conflict, relationship dissolution, violence and were consistent barriers across studies (5, 12, 23).

A Zimbabwe study reported on the fear of reputational damage, and the possibility of violence, both emotional and physical, as a result of notifying partners. Additionally, participants who were not married were afraid of being accused of engaging in premarital sexual activities (12). Other reasons cited for failing to notify partners are related to the logistical challenges surrounding a relationship, namely mobility of partners, not knowing how to contact partners, and partners not living near the index clients (5).

### 6.5 Facilitators of partner notification

The facilitators to PN ranged from method or approach of notification, integrated approach of services and characteristics of diagnosed STI patients. In a study conducted with youth in Zimbabwe, 41% of young people accepted referral slips as a method of PN, though only 5.7% of partners were reported as being treated (12). Similarly, a study reported health care workers’ support and the issuance of referral slip enabled them to notify partners of youth notifying their partners in Zimbabwe (23).

### 6.6 Uptake of treatment following partner notification

Partner notification does not necessarily translate to partner treatment. There was varying uptake of treatment by PN following notification in the studies that were reviewed. Despite strong youth willingness, or recognizing the importance of PN, actual partner treatment uptake was much lower. Reported PN rates ranged from moderate to high across populations in three studies that were reviewed, while partner treatment uptake was under 40% (5, 12). Another study in South Africa, which reports that 74% of youth notified partners, confirms that only 35% of partners received treatment (5).

## 7 Discussion

The review examined evidence on youth, drawing on studies conducted in Zimbabwe and South Africa. The findings in three studies included revealed insights into the suitability of PN strategies for young people aged 15–24 years. The barriers reported to be encountered by youth were fear of partner reaction such as blame, conflict, relationship dissolution and violence (5, 12, 23).

The similar findings were documented in the systematic literature review which aimed to systematically and critically evaluate the STI disclosure which reported that fear was a prominent emotion expressed in other studies (25). Other study in a similar setting to review, exploring the challenges experienced by adolescent girls and young women (AGYW) described that the participants were not willing to notify their partners due to experiencing apprehension (26).

Furthermore, the findings from the three studies reported on role of relationship dynamics in notifying the partners. The casual or steadiness of the relationship were found to influence PN among partners (5, 12). The review divulged that casual nature of relationship heightened non-obligation of the STI diagnosed patients to tell their partners about infection (5, 12, 23). Studies involving pregnant mothers report that they often disclosed their STI status to supportive partners (13, 27) Moreover, notification of casual partners was less frequent because of concerns around reputation, and limitations surrounding contacting the partner (28). In comparison of a study conducted in South Africa which explored PN and STI patient experiences among clinic attendees, participants who were in casual or one-night stand relationship had different challenges in notifying partners about the STI. In this kind of relationship, the participants were not willing to notify their partners. Due to the short-term nature of relationship (10). However, in other settings findings that emerged were that the likelihood of notifying partner(s) was 1.79 times higher for students with multiple sexual partners than for students who had only one partner (15). On contrary, a study conducted among pregnant women in South Africa reported PN is easier if partners were not in concurrent partnership (16).

Review further found that traditional PN that rely on direct communication between partners to disclose diagnosis of STIs poses challenges among youth (12). Beyond the patient related factors that affect notification, the health care system factors played a role on PN. The limited support received by participants in the review contributed to non-disclosure of STI (12). However, the other study's findings in the review revealed that support from health care workers and the issuance of PN slips influenced the youth's ability to inform their partners about potential STI exposure (23). The findings mirror review findings conducted before, which report the importance of coupling the expedited partner therapy with interactive counselling.

While willingness to inform partners was reported to be high, the actual proportion of partners treated was inconsistent, thereby revealing the impact of relationship type, notification method, and gender dynamics on notification (5, 12). The gap between willingness and implementation mirrors the reports from other African and global settings, where PN acceptability exceeded the PN uptake (30, 31).

## 8 Strengths and limitations

This paper systematically synthesizes findings related to youth experiences and practices related to PN of STIs in sub-Saharan Africa. Though earlier reviews have been conducted, they examined HIV disclosure and comprehensive adolescent sexual and reproductive health in sub-Saharan Africa; evidence is limited on examining youth experiences and practices regarding PN among youth. However, this study offers consolidated evidence-based insights on PN that could not be found or presented in any single existing publication. The studies included in this review are heterogeneous and had been undertaken in low–and middle-income countries in the sub-Saharan region, where there was limited data on PN among youth (5, 12, 23). Additional strength lies in methodology, where the selection of studies was conducted independently by three members.

The scoping review is not without limitations. Firstly, studies included were concentrated geographically mainly in Zimbabwe and South Africa, which has a risk of potential publication bias. The concentration of the review in a few countries means that findings may not adequately reflect the experiences of youth in other countries, where there may be different experiences and practices for youth on partner notification.

Another limitation relates to the methodological heterogeneity of the included studies. The studies differed in design, sample size, and data collection approaches, thus limiting the ability to make a direct comparison across the three studies. These limitations collectively affect the generalizability of the review findings. The small number of studies, geographical concentration, and methodological variation mean that the findings must be interpreted cautiously and may not be representative of the youth aged 15–24 years in other countries. Nevertheless, the review provides the important preliminary mapping of available evidence and highlights critical gaps that require research to inform PN strategies for youth in the region.

## 9 The magnitude of the evidence gap

The scoping review revealed an evidence gap on youth experiences and practices of PN for curable STIs. Though only three studies met the eligibility criteria for inclusion, they highlight important barriers for youth (5, 12, 23). The limited number of studies is concerning, given the STI burden among youth 15–24 years of age, and partner notification is recognized as the critical strategy for interrupting the transmission chain for STIs (2–4). However, findings suggest that the experiences, practices, challenges, and facilitators associated with PN among youth remain insufficiently explored by the literature in Sub-Saharan Africa.

## 10 Future research priorities

The evidence on youth's experiences and practices of PN for curable STIs is limited. The evidence highlighted hesitancy among youth to notify partners because of structural, partner, and client context factors. Future research is required to investigate the appropriateness of current PN approaches, the development of safe notification strategies, and to evaluate innovative models for notifying partners for youth 15–24 years of age in sub-Saharan Africa. Additionally, future research must explore how factors such as gender norms, relationship power dynamics, and cultural context influence youth's willingness and their ability to notify their partners about STIs so that evidence would inform PN approaches in similar settings.

The review highlights the need for youth-focused partner notification strategies within the STI prevention programs. Based on the unique vulnerabilities and social circumstances faced by youth, research focusing on tailored youth interventions remains critical in informing youth-friendly health services. The literature points out that PN is suitable for control of STI However, PN did not translate into partner treatment uptake. Therefore, there is a need for research exploring the PN pathway from initial partner mention to treatment initiation. So that findings can inform interventions that support a full cascade from testing to treatment.

The studies report on different barriers to PN. Including the health system constraints, strategies such as task shifting, integration of PN into routine prevention intervention services should be examined to determine ways of strengthening health system structures and support for effective PN implementation.

## 11 Implications for public health policy

Policy makers and public health practitioners requires context specific evidence to inform program designs, implementation, and monitoring. The studies included in the review provide preliminary evidence on curable partner notification amongst youth, underscoring the need for greater investment in research on youth sexual health, development of more responsive and youth-sensitive public health policies, and strengthening reproductive health services to be youth-responsive.

## 12 Conclusion

Partner notification remains a cornerstone for STI control. However, the review reflected that PN remains complex among youth. While evidence points to support for youth contributing to PN, it identified that specific barriers remain for youth partner notification. Moreover, PN willingness does not automatically translate to partner notification and partner treatment. Additionally, there is a need for partner notification strategies that are tailored for youth.
