# Supplementary material for: Mapping evidence on youth experiences and practices on partner notification for sexually transmitted infections in sub-Saharan Africa: scoping review
Source: Front Reprod Health. 2026 Apr 20;8:1729464. doi: 10.3389/frph.2026.1729464 (PMC13136240; doi:10.3389/frph.2026.1729464)
Supplement: Supplementary file 2 [file Table2.docx]

**Appendix D: Reviewed articles (n = 3) published 2016–2026**

| Authors, country, publication date | Aim/Purpose | Population and sample size | Methods | Outcome details, recommendations for future research | Key findings |
| --- | --- | --- | --- | --- | --- |
| Mackworth-Young et al | To explore uptake of STI screening, treatment and PN within a sexual and reproductive health service | 15  Young people aged 16-24 | Qualitative, Intervention | The youth friendly services facilitate uptake of STIs services however research is required on socially safe partner notification strategies. | Experience: young people accepted STI screening and came back for their STI test results because of STI information provided in a friendly manner by health care workers.  The youth were motivated to seek health services by experiencing STI symptoms and health being a priority.  Youth in secure relationships felt able to inform their partners about their STI diagnosis. |
| Chitneni et al.  2020  South Africa | To assess the STI care cascade across participant diagnosis, STI treatment, partner notification and partner treatment  To index recurrent STIs and associated factors and reasons for notifying partner of STI | 216  Youth aged 16−24 years | Prospective cohort | Partner notification by youth is challenging and novel strategies are required to overcome barriers | All survey participants reported completing STI treatment (100%), 17/23 (74%) notified a partner, and 6/23 (35%) reported partner treatment. Overall, 4/23 (11%) participants had 12-month recurrent *C. trachomatis* infection, with no association with partner notification or treatment. STI prevalence was reported at 17%  Reasons for not notifying a partner of STI: Embarrassment, fear of partner judgment, unaware their infection was STI and requiring partner notification, and dissolution of the partnership  Gap: Understanding how to ideally implement STI partner notification by adolescents and youth, especially in a context where point-of-care-based STI diagnostics exist |
| Lariat et al.  2023  Zimbabwe | To understand young people's experiences of partner notification, particularly risks and challenges encountered during patient referral | 1,807  Young people aged 16−24 years | Mixed method | There is a need to interrogate whether partner notification is suitable for youth | Partner notification slip uptake was 41.2%; 5.7% partners returned for treatment  Experiences: Partner notification was framed as a vital element of the treatment pathway  Counselors did not prepare clients for multiple steps of partner notification  Participants reported feeling ill-equipped to counsel and convince their partners to seek treatment  Young people found that partner notification involved unavoidable risk (reputational damage, physical violence, and emotional and relational damage). Youth feared how their partners would react and assumed it would involve blame and spreading rumors that could reach their friends and communities  Young people reported the perceived disconnect between the request to notify partners and the reality of doing so; they referred to the difficulty of notifying partners  Young people refrained from sharing their concerns about partner notification with providers because partner notification was framed as their responsibility to prevent further infections  Youth lost control of information about their STI once partner notification had been done  Partner notification involved dilemmas relating to balancing the risks and telling partners  Gender power dynamics left women being blamed for the STI infection  Youth experienced pressure to notify partners, and assumed that there were no alternatives to notifying partners  Gap: Is partner notification fit for youth or are there alternative interventions appropriate for partner notification which balance risks and protect young people and their engagement in care? |
